# Case Report: Clinical delineation of *CACNA1D* mutation: New cases and literature review

**DOI:** 10.3389/fneur.2023.1131490

**Published:** 2023-04-14

**Authors:** Alshaimaa Alzahrani, Maha Alshalan, Mohammed Alfurayh, Abdulaziz Bin Akrish, Najlaa A. Alsubeeh, Fuad Al Mutairi

**Affiliations:** ^1^Genetic and Precision Medicine Department, King Abdullah Specialized Children Hospital, King Abdulaziz Medical City, Ministry of National Guard Health Affairs (MNGHA), Riyadh, Saudi Arabia; ^2^College of Medicine, King Saud Bin Abdulaziz University for Health Sciences, Riyadh, Saudi Arabia; ^3^King Abdullah International Medical Research Center (KAIMRC), King Saud Bin Abdulaziz University for Health Sciences, Ministry of National Guard Health Affairs (MNGHA), Riyadh, Saudi Arabia

**Keywords:** case report, *CACNA1D* gene, autism (ASC), pervasive development disorder, childhood neurologic disorders, seizure, intellectual disability

## Abstract

**Background:**

Calcium ions are involved in several human cellular processes; nevertheless, the relationship between calcium channelopathies (CCs) and autism spectrum disorder (ASD) or intellectual disability (ID) has been previously investigated. We delineate the spectrum of clinical phenotypes and the symptoms associated with a syndrome caused by an inherited gain-of-function mutation in *CACNA1D* in a family with a history of neuropsychiatric disorders. We also review the clinical and molecular phenotype of previously reported variants of *CACNA1D*.

**Case presentation:**

We report the case of a 9-year-old female patient, diagnosed with ASD, severe ID, hyperactivity, and aggressive impulsive behaviors. The father, who was a 65-year-old at the time of his death, had ID and developed major depressive disorder with catatonic features and nihilistic delusion, followed by rapidly progressive dementia. He died after experiencing prolonged seizures followed by post-cardiac arrest. The patient’s sister was a 30-year-old woman, known to have a severe ID with aggressive behaviors and sleep disorders. The sister has been diagnosed with bipolar disorder and psychosis. Through whole exome sequencing, a heterozygous previously identified and functionally characterized missense likely pathogenic variant was identified in the *CACNA1D* gene NM_001128840.3: c.2015C > T (p.Ser672Leu). These findings are consistent with the genetic diagnosis of autosomal dominant primary aldosteronism, seizures, and neurological abnormalities. This variant was found in the heterozygous status in the patient, her father, and her affected sister.

**Conclusion:**

This case report will help to determine the key clinical features of this syndrome, which exhibits variable clinical presentations.

## Introduction

Autism spectrum disorder (ASD) is a group of conditions in which the person experiences significant difficulties in socialization, communication, and language (1). In addition, people with ASD exhibit unusual repetitive behaviors (2). Despite the lack of a clear mechanism, genetics have a significant role in the etiology of ASD; previous research studies conducted on families and twins have elucidated specific genetic causes for isolated cases, and a number of candidate genes and chromosomal regions have been indicated as relevant across multiple studies, including 2q, 7q, 15q, and 17q (3, 4). Genome-wide studies using genotyping microarrays, whole-exome sequencing, and whole-genome sequencing have identified a rapidly growing number of genes linked to ASD; furthermore, cumulative discoveries of *de novo* mutations have pointed the field in various directions, including the converging actions of ASD-associated mutations and progressive use of translational strategies (in animals and humans) to understand the outcomes of replicated ASD-associated mutations (5–7).

Calcium ions are involved in several human cellular processes including corticogenesis, transcription, and synaptogenesis and play a major role in the development of neurodevelopmental disorders (8). Since the calcium signaling pathway was recognized among the top three pathways involved in ASD, many discoveries of point mutations in the α1-subunit of L-type calcium channels (CaV1.2) and many other genes encoding voltage-gated calcium channel (VGCC) subunits, such as *CACNA1B*-F encoding pore-forming subunits, *CACNB1* and *CACNB2* encoding auxiliary β-subunits, and *CACNA2D3* and *CACNA2D4* for auxiliary α2-δ-subunits have been associated with ASD (9).

Voltage-gated calcium channels (VGCCs) are transmembrane proteins that are activated by depolarization, triggering Ca^2+^ influx into neurons and other excitable cells, which are composed of a pore-forming α1 subunit and auxiliary α2δ and β subunits (10). Until now, 10-mammalian VGCCs have been identified, and based on their activation threshold. CaVs are classified as high or low voltage activated (HVA and LVA) (11). One of the three main families of the LVA subgroup is the L-type Ca^2+^ channels (LTCCs; Cav1), which consists of four subtypes: Ca_v_1.1, Ca_v_1.2, Ca_v_1.3, and Ca_v_1.4 (12). The main functions of Ca_v_1.1 include the excitation–contraction coupling in skeletal muscles and the regulation of transcription in these muscles. The main functions of Ca_v_1.2 include excitation–contraction coupling in cardiac and smooth muscles, hormone secretion, and neurotransmitter release. The functions of Ca_v_1.3 are similar to those of Ca_v_1.2, with the addition of cardiac pace-making and auditory transduction. The Ca_v_1.4 plays a role in visual transduction. Ca_v_1.2 and Ca_v_1.3 are expressed in the most electrically excitable cells, including various areas of the brain (13, 14) (Table 1). The variants in *CACNA1C*, *CACNA1D, CACNA1F*, and *CACNB2* cause gain-of-function by preventing voltage-dependent inactivation of their subunits (Cav1.2, Cav1.3, Cav1.4, and Cavβ2), leading to an excessive influx of Ca^2+^, alteration in kinetics (activation and inactivation), and dominant-negative effects of truncated forms of alpha1 subunits (8). Variants located in *CACNA1A* and *CACNA1H* are the cause of the loss of function in encoded subunits (Cav2.1 and Cav3.2), resulting in decreased channel activity (16).

**Table 1:** Voltage-gated Ca^2+^ channels: classification and human genetic disease.*

Family		Gene	Protein	Clinical phenotype
HVA	Cav1	*CACNA1S*	Cav 1.1	Hypokalemia periodic paralysis type 1 Malignant Hyperthermia type 5 Thyrotoxic periodic paralysis type 1
		*CACNA1C*	Cav 1.2	Timothy syndrome Burgada syndrome type 3 Long QT syndrome type 8
		*CACNA1D*	Cav 1.3	Sinoatrial node dysfunction and deafness Primary aldosteronism, seizures, and neurologic abnormalities
		*CACNA1F*	Cav 1.4	Aland Island eye disease Cone-rod dystrophy, X-linked type 3 Congenital stationary night blindness type2
	Cav2	*CACNA1A*	Cav 2.1	Developmental and epileptic encephalopathy 42 Episodic ataxia type 2 Familial hemiplegic migraine type 1, with or without progressive cerebellar ataxia Spinocerebellar ataxia type 6
		*CACNA1B*	Cav 2.2	Neurodevelopmental disorder with seizures and nonepileptic hyperkinetic Movements
		*CACNA1E*	Cav 2.3	Developmental and epileptic encephalopathy type 69
LVA	Cav3	*CACNA1G*	Cav 3.1	Spinocerebellar ataxia type 42 Spinocerebellar ataxia 42, early-onset, severe, with neurodevelopmental deficits
		*CACNA1H*	Cav 3.2	Hyperaldosteronism, familial, type 4 Childhood absence epilepsy
		*CACNA1I*	Cav 3.3	Not reported

HVA, high voltage activated; LVA, low voltage activated. * The table taken and modified from reference (15).

### Genetic associations between CaVα1 genes and autism spectrum disorders

In total, 10 genes encode the CaVα1-pore-forming subunit of CaVs (CACNA1). Variations in *CACNA1* genes were found to be associated with a variable degree of ASD and other neurodevelopmental disorders. Most variants exhibited a gain-of-function effect. Generally, severe to profound ID/GDD was observed more for the cases with gain-of-function variants as compared to those with loss-of-function. Specifically, *CACNA1E, CACNA1G, CACNA1F, CACNA2D2*, and *CACNA1A* were associated with more severe phenotypes (8).

Based on their pharmacology and sequence similarity, CaVα1 subunits are subdivided into three subfamilies (CaV1, CaV2, and CaV3) (17). The *CACNA1D* gene encodes CaV1.3. This channel contributes to the rhythmic activity of the sinoatrial node and is thereby involved in the regulation of heart rate (18). Disease-causing variants in the *CACNA1D* gene have been associated with autosomal dominant primary aldosteronism, seizures, and neurologic abnormalities (OMIM 615474); and autosomal recessive sinoatrial node dysfunction and deafness (OMIM 614896). Similarly, individuals with variants in the *CACNA1D* gene have been revealed among individuals with ASD (19). Symptoms range from ASD with intellectual disability to seizures, muscle hypotonia, and global developmental delay in more severely affected patients. Some patients also exhibit additional endocrine symptoms (primary aldosteronism or hyperinsulinism) due to the expression of Ca_v_1.3 in adrenal zona glomerulosa cells and pancreatic β-cells. The functional analysis of these reported variants has shown typical gain-of-function changes (20).

In this article, we present a previously reported variant of *CACNA1D*. We delineate the spectrum of clinical phenotypes and the symptoms associated with a syndrome caused by the inherited *CANCA1D* missense mutation in a family with a history of neuropsychiatric disorders.

## Case presentation

The subject of the case report is a 9-year-old girl who was born at full term *via* normal spontaneous vaginal delivery to a healthy mother following an uneventful pregnancy. The neonatal history was unremarkable. Her mother started to seek medical advice at the age of 4 years as the child exhibited speech and language delay with challenging behaviors. These challenging behaviors took the form of aggressiveness, self-injurious behaviors, stereotyped and repetitive motor mannerisms with hand flapping and body rocking, lack of sleep, and attention-deficit hyperactivity-like disorders.

### Developmental history

There were not any concerns regarding her gross motor skills; she sat at 7 months and walked at 14 months and continued to be appropriate for her age in all stages. Her fine motor skills were delayed, she could only hand grasp a pen for scribbling and was not able to dress with a significant stereotypic movement of her hands. Her cognitive and language skills were significantly delayed, she could count from 1 to 10, and she said repeated non-comprehensive limited words and sentences. In the development clinic, she was diagnosed with ASD and severe intellectual disability at the age of 6 years based on DSM-5 criteria. The assessment revealed deficits in social–emotional reciprocity, in nonverbal communicative behaviors used for social interaction, and in developing and maintaining relationships. The assessment also revealed that she had repetitive speech and motor movements, usually with her hands. On examination, her growth parameters were within normal percentiles, and she exhibited no gross dysmorphic features, normal neurological status, and no evidence of cardiovascular abnormalities. Her blood pressure was normal, but she was agitated and hyperactive and made no eye contact during the examination. At the age of 6 years, she was started on risperidone 0.5 mg daily and gradually increased to twice per day, which did not show significant improvement. Later at the age of 8 years, she was started additionally on methylphenidate 5 mg twice per day, which led to little improvement in sleep patterns and no change in hyperactivity or aggressive impulsive behaviors.

### Family history

The parents were non-consanguineous. The mother is 50 years old, and she is not known to have any chronic medical illnesses. The father died at the age of 65 years. Although he was independent in activities of daily living, he was illiterate and was diagnosed to have ID in the form of impaired executive functions. He did not have any behavioral disorders and showed good interpersonal communication. He was diagnosed with hypertension and type 2 diabetes and developed stage 3 chronic kidney disease and coronary artery disease that required percutaneous coronary intervention. In the last 6 months of his life, he developed seizures that were controlled with levetiracetam 250 mg twice per day. All his neuroimaging was unremarkable, including magnetic resonance imaging (MRI) of the brain and computed tomography (CT) angiography of the brain. After 3 months, he started to have mood disturbances and became isolated, with a lack of sleep, loss of appetite, and suicidal ideation but no suicide attempt. An adult mental health team diagnosed him to have a major depressive disorder with catatonic features and nihilistic delusion, followed by rapidly progressive dementia, where he was started on sertraline 25 mg daily. He died after suffering prolonged seizures followed by post-cardiac arrest the next day.

The patient has six siblings, all of whom except one have no medical concerns. She is a 30-year-old female patient who was diagnosed to have severe ID with aggressive behaviors and sleep disorders. At the age of 14 years, she was diagnosed to have bipolar disorder and psychosis, as she has visual and auditory hallucinations, she was given citalopram 2 mg and quetiapine 200 mg daily with a poor response. Her symptoms have currently improved on aripiprazole 5 mg daily and olanzapine 5 mg once before sleep time.

### Laboratory investigations

Laboratory investigations for the patient, including basic and neurometabolic workups, chromosomal analysis, and array comparative genomic hybridization, were unremarkable. A hearing test returned normal results. WES was performed for molecular diagnosis using standard methods.

## Materials and methods

### Clinical data and ethical compliance

Informed consent for genetic analysis, clinical data, and images was obtained from the family, and it was secured at King Abdulaziz Medical City. Clinical investigations were conducted according to the declaration of Helsinki. Genetic analyses were performed in the context of the clinical genetic program by experienced clinical geneticists. Clinical data, pictures, DNA specimens, and other biological materials were collected, used, and stored after signed informed consent was obtained from the participating subjects/families.

### Genomic analysis

Blood samples were collected from the two sisters and parents in EDTA tubes, and Genomic DNA was extracted from the stored EDTA peripheral blood using the QIAampDNA mini kit (Qiagen®) following the manufacturer’s instructions. The final genomic DNA quantity and integrity were performed using NanoDrop 2000 (Thermo Fisher Scientific, Waltham, MA, United States) and Qubit® 3.0 Fluorometer (Thermo Fisher Scientific) following the manufacturer’s protocol. We then proceeded with whole exome sequencing (WES). Double-stranded DNA capture baits against approximately 36.5 Mb of the human coding exome (targeting >98% of the coding RefSeq from the human genome build GRCh37/hg19) were used to enrich target regions from fragmented genomic DNA with the Twist Human Core Exome Plus kit. The generated library was sequenced on an Illumina platform to obtain at least 20x coverage depth for >98% of the targeted bases. An in-house bioinformatics pipeline, including read alignment to GRCh37/hg19 genome assembly, variant calling, annotation, and comprehensive variant filtering, was applied. All variants with minor allele frequency (MAF) of less than 1% in the gnomAD database and disease-causing variants reported in HGMD® in ClinVar were considered. The investigation for relevant variants was focused on coding exons and flanking +/−20 intronic nucleotides of genes with clear gene-phenotype evidence. All potential modes of inheritance patterns were considered. In addition, provided family history and clinical information were used to evaluate identified variants with respect to their pathogenicity and causality. Variants were categorized into five classes (pathogenic, likely pathogenic, VUS, likely benign, and benign). All variants related to the phenotype of the patient were reported. The laboratory had established stringent quality criteria and validation processes for variants detected by NGS. Variants with low quality and/or unclear zygosity were confirmed by orthogonal methods. Consequently, a specificity of >99.9% for all reported variants was warranted.

## Results

### Molecular investigations using WES

To identify the genetic cause of the disorder in the patient and her affected family members, we performed quad whole-exome sequencing, including the index patient, the parents, and the affected sister using standard methods.

### Variants filtration

WES revealed a heterozygous previously identified and functionally characterized missense likely pathogenic variant in the *CACNA1D* gene NM_001128840.3:c.2015C > T (p.Ser672Leu). This result was consistent with the genetic diagnosis of autosomal dominant primary aldosteronism, seizures, and neurological abnormalities. This variant was also found in her father and in her affected sister in heterozygous status but was absent in her healthy mother. No other variants that could explain the patient’s phenotype were detected. The variant was classified as likely pathogenic according to American College of Medical Genetics (ACMG) guidelines.

This variant has previously been described by Hofer et al., who provided evidence that gain-of-function *CACNA1D* mutations, such as S672L, but not loss-of-function mutations, such as S672W, were associated with a high risk of neurodevelopmental disorders including autism (20). As the variant has been previously associated with the disease phenotype, this was likely the correct molecular diagnosis.

### Sanger sequencing

The identified variant (c.2015C > T; p.Ser672Leu) was Sanger sequenced in all members of the family using standard methods. The variant segregated perfectly with the disease phenotype within the family.

### Variant pathogenicity

This substitution (c.215C > T; p.S672L) was predicted to be deleterious by several online computational prediction tools (Mutation Taster; Sorting Intolerant from Tolerant). Further segregation analysis of other siblings supported the pathogenicity of this variant. The identified variant was not observed in the ExAC or gnomAD database, thus confirming the pathogenic nature of the variant.

## Discussion

Previous studies have linked germline *CACNA1D* gene mutations in extended clinical phenotypes with certain neuropsychiatric and neurodevelopmental disorders, childhood epilepsies, and ASD (21). Heterozygous missense gain-of-function mutations are associated with neuropsychiatric disorders, developmental delay, and ASD. Gain-of-function variants in the *CACNA1D* gene have been identified as somatic mutations in aldosterone-producing adenoma (22). These findings support the idea that homozygous loss-of-function mutations, such as frameshift, premature stop codon, and splice-donor defect, result in a loss of channel function that causes deafness, bradycardia, and sinoatrial node arrhythmia (human sinoatrial node dysfunction and deafness syndrome, OMIM 614896) (15).

In this case report, we describe a case that is not related to the previous cases with the S672L variant in *CACNA1D* and with similar phenotypes of ASD (23); however, the patient in this case is reported to have inherited the variant from her father, whose clinical presentation mainly included ID, psychiatric illness, and seizures, which developed near the end of his life, but no autistic features. Moreover, her sister is known to have ID and a psychiatric disorder and exhibits aggressiveness and impulsive behaviors; however, she is not known to have autistic features either. None of the five cases with S672L has hypertension, hyperaldosteronism, or evidence of hyperinsulinemia (Table 2).

**Table 2:** Clinical phenotypes of reported cases with heterozygous missense gain of function mutation of *CACNA1D*.

No	Gender	Variant / Inheritance	Zygosity / Allele Frequencies	Onset	ASD	ID	Seizures	DD	Psychiatric illness	Behavioral	Sleep Disorder	Endocrinological symptoms	Brain MRI	Birth complications	Others	References
1	F	NM_000720.4:c.1208G > A (p.Gly403Asp) *Autosomal dominant*	Heterozygous, gnomAD: *denovo novel variant*	1 month	Unk	+	+	+	Unk	Unk	Unk	Primary aldosteronism	PVL	C/S (36 weeks) Large birth weight	Sinus bradycardia and biventricular hypertrophy, ventricular septum defect, patent foramen ovale; cortical blindness, cerebral palsy; transient hypoglycemia on day 2	24
2	F	NM_000720.4:c.1208G > A (p.Gly403Asp) *Autosomal dominant*	Heterozygous, gnomAD: *denovo Previously reported*	Birth	+	+	+	+	Unk	Unk	Unk	Hypoglycemic hyperinsulinemia	Unk	Large birth weight	Unk	25
3	F	NM_001128840.3:c.2250C > G (p.Ile750Met) *Autosomal dominant*	Heterozygous, gnomAD: *denovo novel variant*	Birth	Unk	+	+	+	Unk	Unk	+	Primary aldosteronism	Normal	C/S (41 weeks) Needs resuscitation	Ventricular hypertrophy; cerebral palsy, spastic quadriplegia, movement disorder with verbal outbursts	24
4	M	NM_001128840.3:c.776 T > C (p.Val259Ala) *Autosomal dominant*	Heterozygous, gnomAD: *denovo novel variant*	1.5 month	Unk	+	+	+	Unk	Unk	Unk	Primary aldosteronism	hypomyelination	UK	Facial dysmorphism, microcephaly	26
5	F	NM_001128840.3:c.2245G > A (p.Ala749Thr) *Autosomal dominant*	Heterozygous, gnomAD: *denovo novel variant*	8 years	+	+	Unk	−	Unk	Unk	+	−	Unk	C/S, floppy infant	Impaired motor skills, clumsy, uncoordinated, night incontinence	27
6	M	NM_001128840.3:c.1219G > A (p.Gly407Arg) *Autosomal dominant*	Heterozygous, gnomAD: *denovo novel variant*	15 years	+	−	−	−	Anxiety, depression	−	Unk	−	Normal	Unk	Unk	15
7	M	NM_001128839.3: c.1201G > C (p.Val401Leu) *Autosomal dominant*	Heterozygous, gnomAD: *denovo novel variant*	4 months	+	+	+	+	Unk	+	Unk	−	Normal	−	Unk	28
8	M	NM_000720.4: c.2015C > T (p.Ser672Leu) *Autosomal dominant*	Heterozygous, gnomAD: *denovo novel variant*	Monozygotic twins present at 13 years	+	+	+	+	Unk	+	Unk	−	−	−	Undescended testes, Facial dysmorphic features	23
9	M				+	+	−	+	Unk	+	Unk	−	Unk	−	Facial dysmorphic features	
10	F	NM_001128840.3:c.2245G > A (p.Ala749Thr) *Autosomal dominant*	Heterozygous, gnomAD: *denovo novel variant*	1 year	+	+	−	+	Unk	+	+	−	Unk	−	Binocular vision disorder	29
11	F	NM_001128840.3:c.812 T > A (p. Leu271His) *Autosomal dominant*	Heterozygous, gnomAD: *denovo novel variant*	Birth	Unk	Unk	−	+	Unk	Unk	Unk	Primary aldosteronism and hypoglycemic hyperinsulinemia	Unk	(32 weeks) maternal preeclampsia	Facial dysmorphic features	30
12	F (Index)	NM_000720.4: c.2015C > T (p.Ser672Leu) *Autosomal dominant*	Heterozygous, gnomAD: *Previously reported, inherited from the father (case 13)*	4 years	+	+	−	−	−	+	+	−	Unk	−	Unk	This review and 23
13	M (Father)			Since childhood	−	+	+ Late in his life	−	MDD, developed in the last 6 months of his life	−	−	−	Normal	−	In the last 6 months in his life, he had rapidly progressive dementia	
14	F (Sister)			10 years	−	+	−	−	Bipolar Visual and auditory hallucination	+	+	−	Unk	−	Unk	

+, present; −, absent; Unk, Unknown; ASD, Autism spectrum disorder; ID, intellectual disability; DD, Developmental delay. M, male; F, female; PVL, periventricular leukomalacia; C/S, caesarean section.

We searched all the studies related to the *CACNA1D* gene that were published in PubMed between 2000 and 2022; detailed information from these articles is organized in Tables 2**,** 3. From the three cases described in this case study and previous clinical reports of 11 individuals, we attempted to delineate the spectrum of clinical phenotypes of missense gain-of-function mutations in the *CACNA1D* gene (15, 24–31).

**Table 3:** Reported clinical phenotypes in patients with *CACNA1D* missense gain of function mutations.

Clinical phenotype	Percentage (%)
Intellectual disability	80%
ASD	55%
Seizures	47%
Developmental delay	60%
Psychiatric illnesses	20%
Aggression/ Self Injurious	40%
Sleep disorders	33.3%
Endocrinological symptoms	33.3%
CHD	13%
Facial dysmorphism	27%

ASD, Autism spectrum disorder; CHD, congenital heart disease.

Most (85%) patients presented with intellectual impairment or disability (only three cases did not report ID, two of which did not detail any clinical phenotype). Over half (55%) of the patients presented with ASD, and it was the presenting clinical phenotype of the subject of this case study (case 12); however, the subject’s father and sister (case 13 and case 14) were not clinically diagnosed to have ASD, which could be related to their late clinical presentation or misdiagnosis of psychiatric illnesses instead of ASD. Aggressiveness and self-injurious behaviors were detected in all cases with the S672L variant except one (case 14). In addition, these phenotypes were detected in two individuals with different variants (A749T and L271H; case 10 and case 12, respectively), and this clinical phenotype accounts for approximately 40% of the reported cases. Endocrine symptoms were not consistent and were only being observed in a third of the cases. They were the first clinical presentation that required immediate therapeutic intervention in patients with primary aldosteronism, seizures, and neurologic abnormalities and in the individuals affected by congenital hyperinsulinemic hypoglycemia (G403D) and (L271H) (24, 25, 30). Their absence does not rule out a *CACNA1D* channelopathy, and their presence cannot be predicted from the variant gating change. Furthermore, they should be monitored for symptoms of primary aldosteronism (case 11) and altered glucose homeostasis even if not present in the peri- and post-natal periods. No clear dysmorphic facial features were reported, and they are not consistent clinical features in the *CACNA1D* gene mutation as they are found in only 28% of the reported cases.

As discussed above, Cav1.3 L-type channels are also present in other tissues. Although the homozygous loss of Cav1.3 function results in congenital deafness and sinoatrial node dysfunction, it is currently unclear whether the heterozygous gain-of-function gating changes of the reported variants result in clinically relevant hearing-related symptoms or directly cause the cardiac abnormalities observed in some cases (15) (Table 2).

Hofer et al. functionally characterized the potential changes in the S652L variant. This mutation significantly shifted the voltage dependence of activation and steady-state inactivation to more negative potentials (~13–17 mV), further increasing window currents at subthreshold voltages. Moreover, it slowed tail currents and increased Ca^2+^ levels during action potential-like stimulations, which is characteristic of gain-of-function changes (20). An understanding of the effects of coding mutations on protein function and interaction can guide the treatment and management strategies. The heterozygous *CACNA1D* variants alter gating changes that increase Cav1.3 channel activity. Thus, the inhibition of Cav1.3 channel activity with drugs appears to be a potential treatment option in the affected individuals. The S672L mutation increased the sensitivity of Cav1.3 to inhibition by the dihydropyridine LTCC blocker isradipine by 3- to 4-fold (20). Although LTCC blockers may normalize the mutation-induced increase in channel function after diagnosis, the mutation may have already caused permanent developmental deficiencies that are resistant to drug treatment, and none of the reported cases showed a conclusive effect of this treatment on neurological or behavioral disorders (24). Therefore, the clinical effect of LTCC blockers needs to be tested in small clinical trials involving affected individuals.

## Conclusion

This case report and all previous clinical reports shed light on the link between the *CACNA1D* gene mutation and neurodevelopmental disorders. A broad spectrum of clinical phenotypes was observed, ranging from ASD, with or without seizures and psychiatric disorders, to severe global developmental delay. The missense gain-of-function mutation in *CACNA1D* is inherited in autosomal dominant mode with complete penetrance, as shown in this case study. The minority of the reported cases have endocrinological disorders, such as hyperaldosteronism and hyperinsulinism, but they should be monitored as they might manifest later in life.

## Data availability statement

The datasets presented in this article are not readily available because of ethical and privacy restrictions. Requests to access the datasets should be directed to the corresponding author.

## Ethics statement

Ethical review and approval was not required for the study on human participants in accordance with the local legislation and institutional requirements. Written informed consent to participate in this study was provided by the participants’ legal guardian/next of kin. Written informed consent was obtained from the individual(s), and minor(s)’ legal guardian/next of kin, for the publication of any potentially identifiable images or data included in this article.

## Author contributions

AA participated in writing the background with significance, case presentations, discussion, conclusion, references, designating tables, and read and approved the final manuscript. MaA, MoA, AB, and NA helped draft the manuscript, read and agree on the final manuscript, and formatted the manuscript according to the journal’s guidelines. FA supervised the overall research, preparing the manuscript reading, and approving the final manuscript. All authors contributed to the article and approved the submitted version.

## Conflict of interest

The authors declare that the research was conducted in the absence of any commercial or financial relationships that could be construed as a potential conflict of interest.

## Publisher’s note

All claims expressed in this article are solely those of the authors and do not necessarily represent those of their affiliated organizations, or those of the publisher, the editors and the reviewers. Any product that may be evaluated in this article, or claim that may be made by its manufacturer, is not guaranteed or endorsed by the publisher.
